# Turning up the heat: Can thermal therapy really protect muscle health in older adults?

**DOI:** 10.1113/EP093373

**Published:** 2025-10-30

**Authors:** Franck Brocherie, Yohan Rousse, Grégoire P. Millet

**Affiliations:** ^1^ Laboratory Sport, Expertise and Performance (EA 7370) French Institute of Sport (INSEP) Paris France; ^2^ University Paris Cité Paris France; ^3^ Institute of Sport Sciences, Faculty of Biology and Medicine University of Lausanne Lausanne Switzerland

1

Sarcopenia (age‐related loss of skeletal muscle mass and function), or dynapenia (low muscle strength or low muscle power) are accompanied by functional decline, exposing older adults to an increased risk of falls and injuries, potentially affecting their quality of life (Araujo et al., 2025). To address it, resistance training and nutrition remain the cornerstone interventions, yet not without difficulties such as comorbidities, injury or low adherence.

In this issue of *Experimental physiology*, heat therapy appears as a promising candidate to counterbalance the ageing‐induced muscle dysfunction. Interestingly, Denny et al. (2025) revamped a previous study showing that contractile characteristics of elderly triceps surae can be improved with a 33‐min leg immersion in water at 33°C, resulting in 20% increase in peak power output, related to a change in the force–velocity relationship of the muscle (i.e. increase in applied force and velocity) (Davies & Young, 1985). This is of primary clinical importance since relative muscle power was recently shown to be a stronger predictor of mortality than relative strength in older men and women.

The authors used also passive localized heating of skeletal muscles (i.e. upper thigh at 50°C; the contralateral limb served as control) for 90 min and assessed contractile function across multiple isokinetic velocities in young versus older adults. Results indicated that a localized muscle heating (+5.3°C without modifying any systemic physiological responses, regardless of age) enhanced peak force and rate of force development (RFD) in both young and older adults, though effects were smaller and less sustained with age. Peak torque increased by ∼8–10% in young and ∼10% in older adults at higher contraction velocities, while RFD rose by ∼25–30%, with relatively greater early‐phase gains in the older adults. As these changes occurred without alterations in EMG amplitude, they likely reflect faster electrochemical and contractile processes rather than neural adaptations.

These findings indicate that heat therapy can enhance contractile performance in aged muscles, potentially supporting functional mobility in the context of reduced neuromuscular drive. This is certainly appealing for geriatric care. First, because the use of localized heat therapy circumvents the potential health risks related to systemic heat exposure‐induced hyperthermia in the elderly population. However, in such intervention, the temperature gradient between muscle and core temperatures tends to diminish, which, with increased intramuscular blood flow, leads to a lower heating impulse. Achieving higher temperatures through diathermy or systemic heat exposure (Watanabe et al., 2024) with (e.g., resistance exercise) or without exercise may elicit greater increases in muscle function, the latter supposedly providing dual benefits regarding the aforementioned muscle‐related improvements and heat acclimation if well‐structured and well‐adjusted heat exposure is guaranteed, alongside careful monitoring. Such potential interaction between passive or active local or systemic exposure to heat deserves further investigation. Second, although the mechanisms underpinning the increase of muscle function following heating remain unknown, the underlying biological/physiological rationale is compelling. The authors have hypothesized an increased blood flow into and out of heated muscle, an improved myofibrillar calcium handling (although the sarcoplasmic reticulum calcium ATPase [SERCA] pump is impaired with ageing), an altered muscle–tendon stiffness, an optimized penetration angle, and an increased ATP turnover and muscle fibre conduction velocity that would further support the increase in RFD and early force production, all favouring postural control, balance and functional mobility in the elderly. At the muscle–tendon level, we would also argue for a faster rate of phosphocreatine utilization, a greater activity of glycolytic enzymes (glycogen phosphorylase, phosphofructokinase and lactate dehydrogenase) and adenine nucleotide degradation, which will contribute to an increased muscle cross‐bridge rate (Brocherie et al., 2024). Such heat‐induced accelerated muscle contraction by shortening electromechanical delay through faster electrochemical processes, thereby enhancing early RFD without altering tendon transmission properties (Mornas et al., 2021, 2022), could be particularly beneficial for older adults, in whom maintaining or restoring RFD is strongly linked to functional capacity, mobility and independence (Aagaard et al., 2010).

In this Viewpoint, we would like to take the opportunity to expand briefly on some possible heat‐related mechanisms that may preserve muscle health in ageing, as well as other environmentally based convergent mechanisms that may complement (or substitute for) heat therapy. Passive heating modalities also induce heat‐shock proteins (HSPs), key molecular chaperones that protect against proteotoxic stress, limit atrophy and support mitochondrial function. Repeated exposures also promote vasodilation and microvascular remodelling, improving oxygen and nutrient delivery to muscle. When combined with exercise, heat amplifies these effects by increasing metabolic stress, activating anabolic signalling cascades (including Akt–mechanistic target of rapamycin and HSP‐dependent pathways) while elevating blood flow and post‐exercise nutrient delivery. In parallel, heat may trigger hormetic antioxidant and anti‐inflammatory adaptations, and enhance insulin sensitivity and glucose uptake, thereby mitigating cardiometabolic risk factors associated with ageing and sarcopenia (Laukkanen & Kunutsor, 2024). Altogether, these combined mechanical, metabolic and thermal stimuli can potentiate muscle adaptation, although robust data are limited.

From an environmental perspective, hypoxic stress activates mechanisms that partly overlap with those triggered by heat therapy, and both may converge on common targets relevant to an enhanced muscle function. Hypoxic stress upregulates hypoxia‐inducible factor‐1α (HIF‐1α), which increases vascular endothelial growth factor expression and promotes angiogenesis favouring tissue perfusion and oxygen transport. Cross‐talk between HIF‐ and HSP‐regulated pathways suggests potential synergy, with both hypoxic and heat stressors converging on shared biological/physiological targets, acting to improve muscle perfusion, oxidative capacity and resistance to muscle atrophy.

Overall, this viewpoint can be regarded as cautiously optimistic as Denny et al. (2025) paved the way for considering heat therapy as an additional (and not a replacement for exercise alone or nutrition) or complementary strategy to sustain muscle health. Given the shared and/or cross‐talk‐based mechanistic promise, this also opens the door for combined or sequential application that could produce additive effects to counteract sarcopenia. The benefits of such a strategy combining different environmental stressors (heat, hypoxia) have been shown recently for the recovery of exercise‐induced muscle damage (Rousse et al., 2025). This warrants further investigations to examine whether heat or hypoxic therapy used alone, combined or in sequence can deliver clinically meaningful improvements in muscle mass, strength and function without increasing physiological risk; and to determine the durability of benefits, the optimal dosing and the safety of repeated exposure in populations with cardiovascular comorbidities. If proven effective, turning up the heat and other environmentally based approaches may one day form part of an integrative strategy to preserve muscle health across the lifespan. Meanwhile, their translation to clinical practice must be approached cautiously, with any therapeutic application carefully individualized, with controlled dosing, monitoring and medical supervision.

## AUTHOR CONTRIBUTIONS

Franck Brocherie conceived the viewpoint and wrote the first draft of the manuscript. Yohann Rousse and Grégoire P. Millet participated in writing and revising the Viewpoint. All authors have read and approved the final version of this manuscript and agree to be accountable for all aspects of the work in ensuring that questions related to the accuracy or integrity of any part of the work are appropriately investigated and resolved. All persons designated as authors qualify for authorship, and all those who qualify for authorship are listed.

## CONFLICT OF INTEREST

None declared.

## FUNDING INFORMATION

None.
